# Metabolic Flexibility as a Candidate Mechanism for the Development of Postoperative Morbidity

**DOI:** 10.1213/ANE.0000000000007494

**Published:** 2025-04-02

**Authors:** Pietro Arina, John Whittle, Maciej R. Kaczorek, Davide Ferrari, Nicholas Tetlow, Amy Dewar, Robert Stephens, Daniel Martin, S. Ramani Moonesinghe, Evangelos B. Mazomenos, Mervyn Singer

**Affiliations:** From the ^1^Bloomsbury Institute of Intensive Care Medicine, Division of Medicine, University College London, London, UK; 2 Department of Medical Physics and Biomedical Engineering, Wellcome/Engineering and Physical Sciences Research Council Centre of Interventional and Surgical Sciences, University College London, London, UK; 3 Division of Surgery and Interventional Science, Research Department of Targeted Intervention, Centre for Perioperative Medicine, University College London, London, UK; 4 University College London Hospitals National Health Service Foundation Trust, London, UK; 5 Department of Population Health Sciences, King’s College London, London, UK; 6 Faculty of Health, Peninsula Medical School, University of Plymouth, Plymouth, UK.

## Abstract

**BACKGROUND:**

This study investigates the role of metabolic flexibility in determining perioperative outcomes. Metabolic flexibility, a key feature of metabolic health, is the ability to efficiently switch between different fuel sources (predominantly carbohydrates and fats) depending on energy demands and availability. Given the rapidly changing physiological conditions in the perioperative period, we hypothesized that good metabolic adaptability could mitigate postoperative complications.

**METHODS:**

We conducted a retrospective observational study utilizing a prospectively collected, single-center preoperative cardiopulmonary exercise testing (CPET) database of patients undergoing a range of major surgeries between 2012 and 2022. On day 3, patients were categorized into 3 groups based on their Postoperative Morbidity Survey (POMS) scores: 0 to 1, 2, and 3 to 6. Metabolic flexibility was evaluated through measurements of fat and carbohydrate oxidation during exercise testing (CPET). Associations were explored between metabolic flexibility, cardiorespiratory fitness, and postoperative outcomes.

**RESULTS:**

Of 585 patients, those with no or low postoperative day 3 morbidity (POMS 0–1; n = 204) demonstrated significantly higher fat oxidation early in exercise before anaerobic threshold (fatty acid oxidation [FATox] area under the curve [AUC] 826 [578–1147]) compared to both POMS 2 (658 [448–922; n = 268]) and POMS 3 to 6 (608 [414–845; n = 113]); both *P* < .001. POMS 0 to 1 patients also had more effective carbohydrate utilization at peak exercise intensity. Higher postoperative morbidity (POMS) categories were associated with diminished metabolic flexibility characterized by a reduced ability to switch between metabolic substrates—carbohydrate oxidation (CHOox) POMS 0 to 1 group AUC 10277 (interquartile range [IQR] 7773–13358) compared to POMS 2 AUC 8356 (IQR 6548–10377) and POMS 3 to 6 AUC 6696 (IQR 473–9392); both *P* < .001. Reduced metabolic flexibility correlated with increased postoperative complications and an extended hospital stay.

**CONCLUSIONS:**

Metabolic flexibility may be a pivotal factor in determining postoperative outcomes. Patients with greater metabolic adaptability had fewer complications and shorter hospitalization by 4 days on average. This suggests that preoperative metabolic conditioning—something potentially achieved by targeted prehabilitation—could be linked to surgical recovery. Future research should focus on prospective studies to confirm these relationships and explore underlying mechanisms. If confirmed, metabolic flexibility assessments could be integrated into routine preoperative evaluation to better predict and improve patient outcomes.

**KEY POINTS**

**Question:** Can metabolic flexibility, the capacity to adapt substrate usage to metabolic demand, as measured by fat and carbohydrate oxidation during preoperative cardiopulmonary exercise testing, predict postoperative morbidity in patients undergoing major surgeries?

**Findings:** Metabolic flexibility was found to be significantly associated with postoperative outcomes, indicating it may be a crucial factor in determining patient morbidity following major surgery.

**Meaning:** Assessing metabolic flexibility preoperatively could enhance the prediction of postoperative morbidity, offering new opportunities for patient optimization and improved surgical outcomes.

More than 320 million surgeries are performed annually worldwide,^1^ in an increasingly aged population.^2^ Perioperative mortality,^3^ currently estimated at 1.7% to 5.7%,^4–6^ accounts for approximately 8 million fatalities annually (7.7% of total global deaths).^7^ Postoperative morbidity, defined as significant deviations in postsurgical recovery, is a significant concern; 14% to 20% of patients currently experience complications with impacts on lifespan and quality of life.^6,8,9^

A challenge for perioperative medicine (POM) is to evolve towards a precision medicine model, in particular identification and targeting of key mechanisms that underlie the development of morbidity. The relationship between reduced cardiorespiratory fitness (CRF) and postoperative morbidity after major surgery is well established.^10^ Low fitness is a modifiable risk factor, and assessment of fitness is an integral part of preoperative assessment. Interventions such as prehabilitation that target fitness show promise in improving perioperative outcomes.^11,12^ Nonetheless, mechanisms through which CRF influences perioperative outcomes are poorly described.

A promising candidate mechanism is metabolic flexibility.^13^ This refers to the capacity to adapt substrate usage (fuel selection—primarily carbohydrates and fats) to metabolic demand and substrate availability,^14,15^ representing an essential mechanism for modulating responses to external stressors such as surgery. Metabolic inflexibility, an impaired ability to switch efficiently between fuel sources, is a pathological state broadly associated with decreased physiological adaptability. It often results in the excessive reliance on 1 energy substrate (eg, carbohydrates in insulin resistance). It is related to both the metabolic syndrome (present in up to 42% of surgical patients)^16^ and reduced CRF.^17^ Persistent carbohydrate oxidation (CHOox) during fasting (reduced lipid oxidation), inadequate shifts to carbohydrate metabolism in the post prandial state or during metabolic stressors such as exercise are hallmarks of the condition. Insulin resistance is a key component of metabolic inflexibility and the metabolic syndrome, resulting in hyperglycemia, impaired mitochondrial fatty acid oxidation and excess accumulation of lipid metabolites. This is associated with higher rates of perioperative infectious, cardiovascular, pulmonary, and renal morbidity.^16^ Metabolic flexibility is a promising target for prehabilitation since regular aerobic exercise improves mitochondrial efficiency, thereby increasing conditional fatty acid and glucose oxidation at times of increased oxygen demand.^17^

Metabolic inflexibility may be implicated in the development of postoperative complications through various mechanisms. These include a potential inability, in the context of increased postoperative metabolic demand and/or relative glycogen depletion, to access stored fuels such as lipids,^13^ alterations in hemostasis and atherosclerotic plaque stability,^16^ and alterations in the immune/inflammatory response to stress.^17^

Exercise requires metabolic flexibility to match fuel availability and substrate selection with enormous increases in metabolic demand. During low-intensity exercise, fatty acid oxidation increases to a maximum rate. In parallel, glucose metabolism increases, ultimately becoming the primary energy substrate via intermediary aerobic glycolysis and then anaerobic glycolysis at maximal exercise. Individuals with higher CRF can use more lipids for longer at lower exercise (and hence metabolic) intensities and use more glucose at higher exercise intensities.^14^

Cardiopulmonary exercise testing (CPET), which uses the same principles as indirect calorimetry to assess oxygen consumption and carbon dioxide production, is an effective method for assessment of both CRF in the perioperative period and for substrate selection during exercise.^14^ Oxygen consumption and carbon dioxide production can, via standard stoichiometric equations, be used to derive fat and CHOox during exercise, and this can be graphed as a metabolic crossover plot.^14^ The measurement of substrate utilization relies on the difference in carbon and oxygen contents of different metabolic substrates; in general, formulas used to estimate usage are based on the respiratory quotient (RQ: carbon dioxide production [VCO_2_]/oxygen consumption [VO_2_]). The RQ ranges from 0.7 to 1.0 in humans; CHO oxidation is greater approaching 1.0 and fat greater approaching 0.7. Substrate selection during exercise differs between elite, recreationally active, and inactive individuals.^14^ It differs between lean, overweight, and obese individuals independently of age, sex, or CRF. Pilot data for this study described similar patterns of substrate selection related to aerobic fitness in patients listed for cystectomy.^18^ Whether these differences are (i) present in a wider population of patients listed for major surgery, and (ii) associated with the development of postoperative morbidity independently of CRF is not known. We hypothesized that metabolic inflexibility, defined using substrate selection during exercise testing, is a candidate mechanism for the development of increased postoperative morbidity. We thus assessed the relationships between CRF, metabolic inflexibility, and the subsequent development of postoperative morbidity and mortality in a large perioperative patient database.

## METHODS

### Population and Selection Criteria

**Figure 1.**
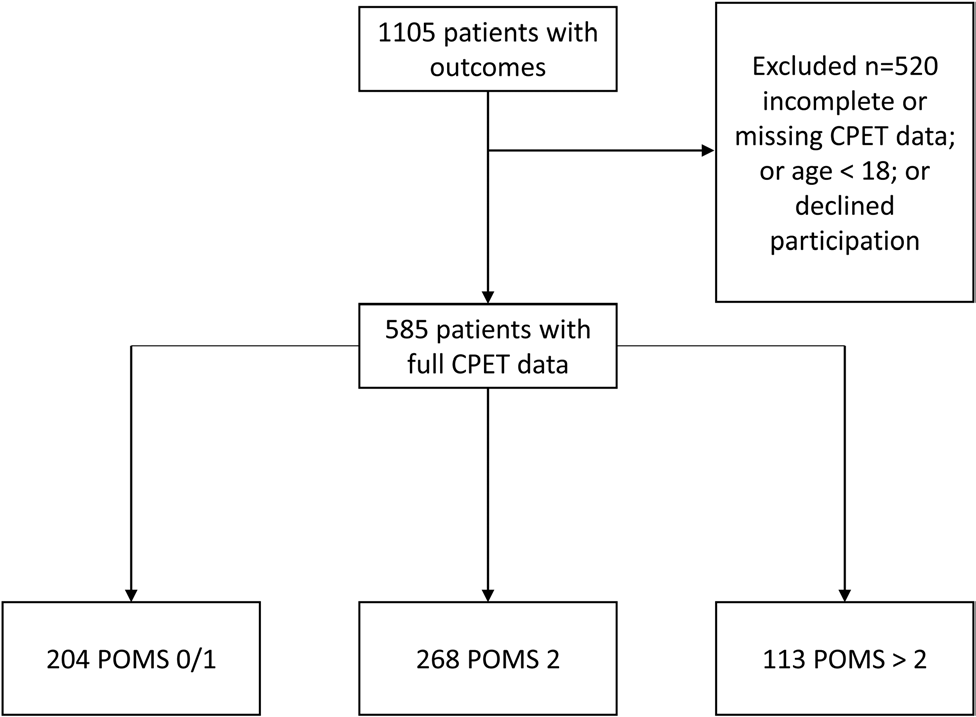
Study flow CONSORT diagram. This diagram illustrates the study flow, detailing inclusion and exclusion criteria, and displaying the final 3 study groups. CONSORT indicates Consolidated Standards of Reporting Trials.

We performed a retrospective observational study of a prospectively collected, single-center, preoperative CPET research database. Patients had given written informed consent for their demographic, CPET, operative, and outcomes data to be stored on an institutional database and to allow subsequent review. The database spans from 2012 to 2022 and includes all patients routinely referred for preoperative CPET across a spectrum of complex major surgeries. The participant selection process is illustrated in the Consolidated Standards of Reporting Trials (CONSORT) flow diagram (Figure 1).

Routine laboratory results were sourced from the University College London Hospitals’ electronic health record system (Epic System Corporation). A rigorous verification process was conducted independently by 2 researchers (M.K. and P.A.) for quality and completeness.

### Cardiopulmonary Exercise Testing

All patients underwent CPET as part of their routine preoperative assessment. Testing was performed in accordance with Peri-Operative Exercise Testing and Training Society (POETTS) guidelines.^19^ A symptom-limited incremental ramp protocol to volitional exhaustion was performed on an electromagnetically-braked cycle ergometer (Lode Corival 906900, B.V. Medical Technology). Breath-by-breath gas-exchange analysis was derived using a volume transducer and a gas sample line attached to a tight-fitting facemask connected to a metabolic cart (Metalyzer 3B, Cortex Biophysik GmbH).

Before each test, the metabolic cart was calibrated in line with the manufacturer’s recommendations and clinical guidelines. A suitable ramp was selected to achieve 8 to 12 minutes of incremental exercise. The ramp protocol selection was based on physiologist experience which considered patient weight, age, sex, current physical activity levels, and recent hemoglobin concentration. The patient was instructed to maintain a constant cadence of 60 rpm. The exercise protocol consisted of 3 minutes’ rest, 3 minutes’ unloaded cycling, incremental loaded exercise until volitional exhaustion (between 8 and 12 minutes), and between 3 and 5 minutes’ recovery time, or until near-baseline physiological values were reached.

### CPET Variables

Oxygen uptake (VO_2_) and carbon dioxide production (VCO_2_), respiratory rate, tidal volume, and end-tidal gas tensions (Oxygen: P_ET_O_2_ and Carbon Dioxide: P_ET_CO_2_) were recorded. Ventilatory equivalents for oxygen (V_E_/VO_2_) and carbon dioxide (V_E_/VCO_2_), and the oxygen pulse (VO_2_/HR) were derived. Values for V_E_/VCO_2_ were derived at anaerobic threshold (AT) and VO_2_ peak (defined as the highest average oxygen uptake over the last 30 seconds of ramped exercise). The ventilatory AT was determined using a combination of the V-slope method, changes in ventilatory equivalents, and end-tidal gas tensions as per POETTS guidance.^19^ All tests were independently interpreted by 2 clinical exercise physiologists and further verified by a CPET-experienced consultant anesthetist. Values for peak VO_2_ and AT were adjusted for body mass (mL.min^−^^1^.kg^−^^1^).

### Perioperative Dataset

Patient characteristics: (age, sex, body mass index [BMI]), comorbid illness, American Society of Anesthesiologists (ASA) physical status, Duke Activity Status Index (DASI),^20^ smoking status, type of surgery, postoperative care destination and length of stay (hospital, high dependency unit [HDU]/intensive care unit [ICU], ward). These data points were selected since they are routinely collected in the CPET database examined in this study and represent known associations with perioperative outcomes.

Presence or absence of postoperative complications using the Postoperative Morbidity Survey (POMS): The POMS is a validated tool designed to systematically capture and categorize morbidity in the immediate postoperative period. It is used to assess complications across multiple organ systems, or 9 domains. These domains represent areas of clinical concern postoperatively and include pulmonary, infectious, gastrointestinal, cardiovascular, neurological, wound, pain, renal, and hematological components. The POMS allows systematic tracking of postoperative complications and evaluation of the burden of morbidity and its impact on recovery.^21,22^

Morbidity was described as a dichotomous nonweighted outcome for any positive score in each of the 9 POMS domains on Postoperative day 3.^22^

**Figure 2.**
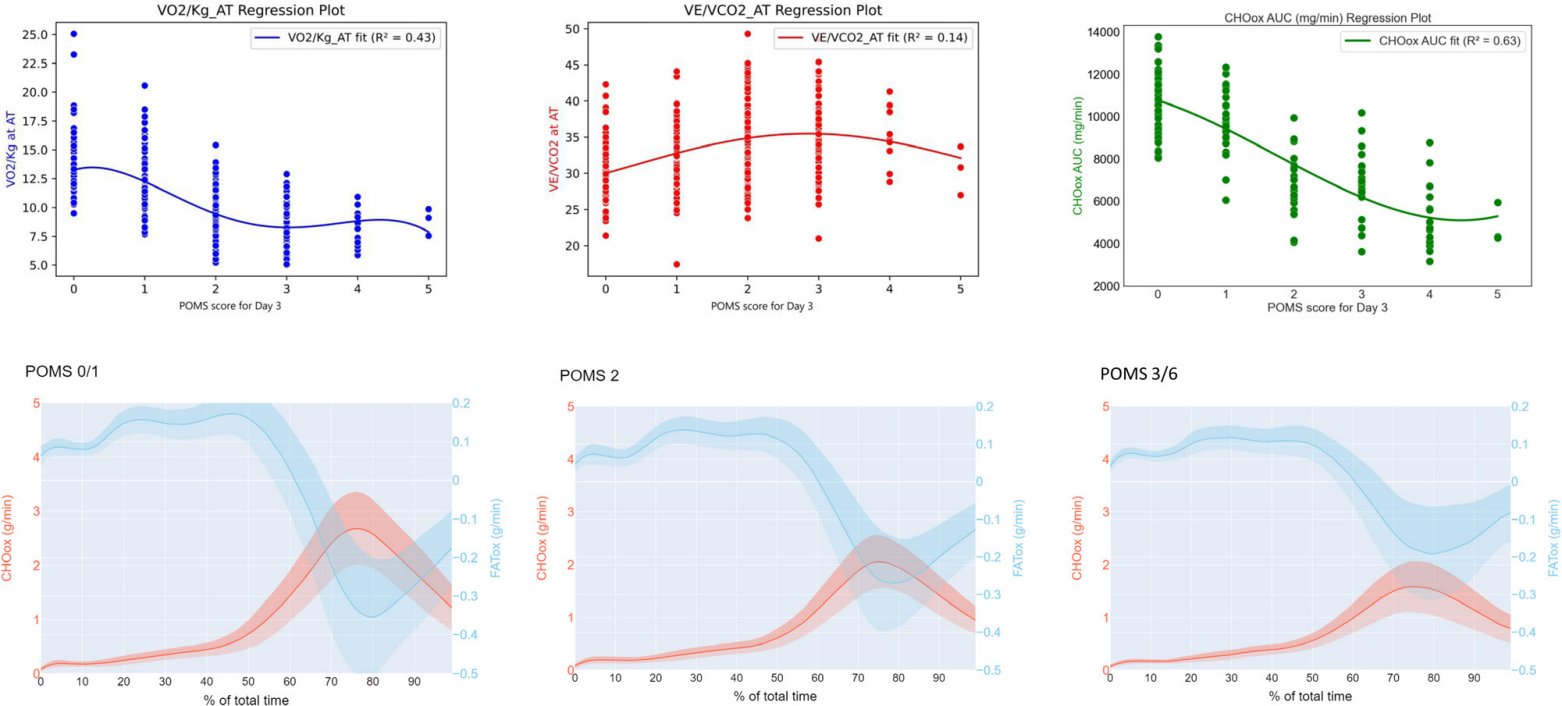
Curvilinear regressions of VO_2_/kg and VE/VCO_2_ and metabolic activity across POMS groups. Upper part on the left, the curvilinear regression of VO_2_/kg at AT (aerobic threshold) over cumulative POMS at day 3 is depicted (*P* < .05, *R*² = 0.43). On the center, the curvilinear regression of VE/VCO_2_ at peak over cumulative POMS at day 3 is shown (*P* < .05, *R*² = 0.14), on the right, the curvilinear regression of CHOox at peak in relation to cumulative POMS on day 3 is displayed (*P* < .05, *R*² = 0.63). Bottom part, 3 panels POMS group: 0 to 1, 2, and 3 to 6, arranged from left to right, depicting the energy expenditure in Kcal/min of CHO and fat for each group with a 95% confidence interval, illustrating metabolic rates. CHO indicates carbohydrate; CHOox, carbohydrate oxidation; POMS, Postoperative Morbidity Score; VCO_2_, carbon dioxide production; VE, minute ventilation; VO_2_, oxygen consumption.

For our analysis, we predefined 3 POMS categories to simplify interpretation and facilitate a clearer comparison of the severity of postoperative complications across different patient subgroups. No patients in our database exhibited more than 6 positive domains on day 3 after surgery; hence, this category was not included (Figures 1 and 2):

**Table 1.** Patient Data Categorized by POMS Groups: 0 to 1, 2, and 3 to 6

	POMS 0–1 median (IQR)—N 204	POMS 2 median (IQR)—N 268	POMS 3–6 median (IQR)—N 113	ANOVA P value	POMS 0–1 vs POMS 2	POMS 0–1 vs POMS 3–6	POMS 2 vs POMS 3–6
Demographics							
Age (y)	65.5 (56–7)	72 (62–78)	74 (65–79)	<.001	++	++	--
BMI (kg/m^2^)	24. 7 (21.6–28.7)	26.8 (23.9–30.8)	27.4 (24.8–31.2)	<.001	++	++	--
Outcome							
LOS (d)	9 (7–15)	11 (8–16)	13 (9–20)	<.001	--	++	++
Score							
ASA	2 (2–3)	3 (2–3)	3 (2–3)	<.001	++	++	--
Duke score	51 (43–58)	45 (31–58)	40 (30–58)	<.001	++	++	--
Lab and BP values							
Creatinine (μmol/L)	80 (67–93)	83 (69–100)	79 (67–91)	.14	--	--	--
Hb (g/L)	133 (120–140)	132 (119–144)	130 (119–140)	.34	--	--	--
Na (mEq/L)	140 (138–141)	140 (138–141)	140 (138–142)	.88	--	--	--
K (mEq/L)	4.3 (4.0–4.5)	4.3 (4.0–4.6)	4.3 (4.0–4.6)	.24	--	--	--
Urea (mmol/L)	6.9 (4.6–7.5)	7.5 (5.7–7.5)	7.2 (5.2–7.5)	.54	--	--	--
White blood cell (x 10^9^/L)	7.9 (6.5–9.2)	8.1 (6.5–9.0)	7.9 (6.8–8.8)	.58	--	--	--
Systolic BP (mm Hg)	134 (123–145)	132 (123–149)	139.00 (121–155)	.28	--	--	--
Diastolic BP (mm Hg)	79 (73–87)	78 (69–86)	82 (72–90)	.08	--	--	--
Mean BP (mm Hg)	97 (90–106)	96 (88–106)	98 (90–109)	.13	--	--	--
CPET—baseline values							
Average time	9 (5–14)	8.5 (4–13)	8 (4–13)	.25	--	--	--
HR (bpm)	84 (75–95)	84 (73–95)	87 (76–96)	.51	--	--	--
VO_2_/HR	3.4 (3.0–4.2)	3.2 (2.7–3.9)	3.2 (2.6–3.8)	.008	++	++	--
VO_2_/kg (mL/kg/min)	4.0 (3.5–4.5)	3.5 (3.0–4.1)	3.5 (3.0–4.0)	<.001	++	++	--
RER	0.83 (0.80–0.86)	0.83 (0.79–0.89)	0.84 (0.80–0.87)	.026	++	--	--
VE/VCO_2_ (mL/min)	39.4 (35.0–44.6)	41.5 (36.9–45.6)	39.8 (35.1–44.7)	.025	++	--	--
CPET—AT values							
HR (bpm)	111 (102–122)	105 (92.8–114.2)	105 (95–116)	<.001	++	++	--
VO_2_/HR	8.3 (6.7–10.0)	6.8 (5.9–8.3)	6.4 (5.1–8.2)	<.001	++	++	--
VO_2_/kg (mL/kg/min)	12.3 (9.2–13.8)	10.3 (8.2–11.2)	10.1 (7.3–11.0)	<.001	++	++	--
RER	0.86 (0.82–0.90)	0.86 (0.81–0.90)	0.85 (0.80–0.89)	.81	--	--	--
VE/VCO_2_ (mL/min)	31.2 (28.7–34.4)	34.9 (32.2–38.1)	34.6 (31.4–37.8)	<.001	++	++	--
CPET—peak values							
HR (bpm)	146 (132–159)	135 (121–150)	135 (119–149)	<.001	++	++	--
VO_2_/HR	10.5 (8.6–12.9)	9.2 (7.3–10.8)	8.0 (6.1–10.6)	<.001	++	++	--
VO_2_/kg (mL/kg/min)	20.3 (15.3–23.7)	15.5 (13.1–17.9)	14.5 (10.7–17.0)	<.001	++	++	--
RER	1.13 (1.05–1.21)	1.16 (1.05–1.24)	1.12 (1.05–1.21)	.055	--	--	--
VE/VCO_2_ (mL/min)	32.4 (29.3–36.1)	35.5 (32.2–38.8)	34.3 (31.6–38.5)	<.001	++	++	--
MET	5.3 (4.4–6.4)	4.5 (3.8–5.4)	4.1 (3.2–5.0)	<.001	++	++	--

Details include demographics (BMI), outcome (LOS), clinical scores (ASA and DASI scores), laboratory test results and blood pressure values (Hb, Na, K, BP), and CPET metrics at different phases (baseline, AT, peak values), HR, VO_2_, RER, VE, VCO_2_, MET). Data are presented as median values with IQR, or as absolute numbers with percentages. The ANOVA *P* value column and the +±- columns in pairwise comparisons indicate the statistical significance of the differences between groups.

Abbreviations: ANOVA indicates analysis of variance; ASA, American Society of Anesthesiologists; AT, aerobic threshold; BMI, body mass index; BP, blood pressure; CPET, cardiopulmonary exercise test; DASI, Duke Activity Status Index; Hb, hemoglobin; HR, heart rate; IQR, interquartile range; K, potassium; LOS, length of stay; MET, metabolic equivalent of task; Na, sodium; POMS, Postoperative Morbidity Score; RER, respiratory exchange ratio; VCO_2_, carbon dioxide production; VE, minute ventilation; VO_2_, oxygen consumption.

- **POMS 0 to 1**: Individuals who experienced no or mild morbidity, with only 1 morbidity domain recorded.
- **POMS 2**: Individuals encountered moderate morbidity, with 2 morbidity domains recorded as positive.
- **POMS 3 to 6**: Individuals developing severe morbidity, with at least 3 and up to 6 morbidity domains recorded as positive.

To facilitate comparison between different durations of exercise, the CPET data for each POM group were normalized to the total time of each CPET session. This was done after confirming no statistical difference existed in absolute test duration (median values: POMS 0–1, POMS 2, and POMS 3–6 groups were 9 [interquartile range {IQR} 5–14], 8.5 [IQR 4–13], and 8 [IQR 4–13] minutes, respectively; *P* = .2450; Table 1).

### Regression Analysis

Polynomial curves were fitted in nonlinear regression analysis between the CPET values and the increasing POMS categories. For each variable, we selected 1 optimal polynomial degree based on a combination of visual assessment, AIC minimization, and cross-validation on weighted mean squared error to balance the model complexity and the fit quality. Moreover, a linear regression analysis was computed and adjusted to account for confounding variables, with a comprehensive list available in Supplemental Digital Content 1, Supplementary Table 1, http://links.lww.com/AA/F256. We did not apply any preprocessing techniques to the variables, sex is represented as 0 for males and 1 for females.

### Metabolic Flexibility

Carbohydrate (CHOox) and FAT (fatty acid oxidation [FATox]) oxidation were determined from VO_2_ and VCO_2_ values measured during CPET. This calculation was performed using standardized stoichiometric equations (nonprotein respiratory exchange ratio [RER]):^14,23–25^


$$\begin{array}{c} CHO\;oxidation\;\left(g/min\right)=4.585\;VCO2\;\left(L/min\right)-3.226\;VO2\;\left(L/min\right) \end{array}$$

$$\begin{array}{l} FAT\;oxidation\;\left(g/min\right)=1.695\;VO2\;\left(L/min\right)-1.701\;VCO2\;\left(L/min\right) \end{array}$$

Total CHOox (carbohydrate) and FATox were subsequently converted to energy consumption measured in calories where carbohydrates and fat produce 4 and 9 kcal/g, respectively.^14^ The reliability and validity of these formulae were confirmed by demonstrating agreement between indirect calorimetry and a reference method for the stoichiometric equations used to estimate FATox and CHOox rates.^26^

### Statistical Analysis

Both descriptive and inferential statistical analyses were conducted using Python (version 3.10.4, Python Software Foundation). Data were first tested for normality with the Shapiro-Wilk test. Depending on the distribution characteristics, values were either reported as median and IQR or as mean and standard deviation. Relative distributions were calculated for categorical values.

The results of the statistical analysis and regression analysis performed in this study were reported after adjusting for the influence of confounding variables including sex, age, BMI, comorbidities, and operation severity.

To investigate differences between the 3 distinct POM groups, statistical tests were used as appropriate including Student’s unpaired *t*-test, Mann-Whitney *U* test, χ^2^ test and 1-way analysis of variance (ANOVA) with Tukey post hoc analysis. A value of *P* < .05 was considered statistically significant.

### Ethics

University College London Hospitals NHS Foundation Trust (UCLH) maintains a prospective research database of patients who underwent CPET before major complex surgery. All participants provided written consent for the inclusion of their CPET results in the database for future research, in accordance with the Declaration of Helsinki. Ethical approval was initially granted in 2012 and reaffirmed in 2019, with no time limitations (NRES Committee London—Southeast reference: 12/LO/0192, London—Westminster Research Ethics Committee reference: 19/LO/1371). The database was designed to facilitate the study of both short- and long-term postoperative morbidity and mortality across a broad spectrum of patients undergoing CPET before complex major surgery. For this study, the database was queried for patients enrolled between 2012 and 2022, with strict adherence to Caldicott principles to ensure data confidentiality and integrity.

## RESULTS

Of the 1105 patients screened, 585 individuals met the inclusion criteria, and their data were consequently analyzed (CONSORT diagram, Figure 1). The POMS 0 to 1 group contained 204 individuals, the POMS 2 group 268 individuals, and the POMS 3 to 6 group 113 individuals.

**Table 2.** Patient Data Categorized by POMS Groups: 0 to 1, 2, and 3 to 6

	POMS 0–1 (n = 204(	POMS 2 (n = 268)	POMS 3–6 (n = 113)	χ^2^ statistic	χ^2^ *P* value
Demographics					
Sex (M/F)	160 (78%)/44 (22%)	186 (69%)/82 (31%)	61 (54%)/52 (46%)	17.31	<.001
Outcome					
30-d mortality	1 (0.5%)	4 (1.5%)	6 (5%)	4.81	.04
1-y mortality	3 (1.5%)	8 (3%)	10 (9%)	8.44	.015
30-d readmission	18 (9%)	15 (6%)	7 (6 %)	2.06	.36
Adverse event	37 (18%)	35 (13%)	22 (19%)	2.35	.31
Past medical history					
Previous MI	42 (21%)	88 (33%)	37 (33%)	8.12	.017
Angina	8 (4%)	5 (2%)	8 (7%)	5.15	.08
Coronary stent	7 (3%)	11 (4%)	8 (7%)	1.79	.41
Previous CABG	3 (1.47%)	8 (3%)	2 (1%)	1.42	.49
Hypertension	61 (30%)	106 (40%)	49 (44%)	5.69	.06
Diabetes	17 (8%)	44 (16%)	23 (20%)	8.41	.015
Cardiac failure	21 (10%)	32 (12%)	20 (18%)	3.56	.17
Peripheral vascular disease	3 (2%)	2 (1%)	3 (3%)	1.05	.59
CVA or TIA	5 (2%)	9 (3%)	5 (4%)	1.05	.59
COPD	14 (7%)	21 (8%)	13 (12%)	1.66	.44
Asthma	14 (7%)	21 (8%)	11 (10%)	0.94	.63
Pulmonary embolism	1 (0.5%)	7 (3%)	0 (0%)	4.53	.10
Pulmonary fibrosis	0 (0%)	1 (0.4%)	0 (0%)	1.15	.56
Smoking	133 (65%)	176 (66%)	66 (58%)	1.54	.46
Arthritis	16 (8%)	40 (15%)	29 (26%)	16.97	<.001
Medications					
Beta blocker	6 (3%)	35 (13%)	18 (16%)	15.93	<.001
Nitrates	6 (3%)	9 (3%)	9 (8%)	4.30	.12
ACE inhibitor	23 (11%)	43 (16%)	18 (16%)	2.03	.36
Statins	36 (18%)	87 (32%)	32 (28%)	11.76	.003
Operation type					
Colorectal	41 (20%)	51 (19%)	23 (20%)	0.95	.67
Upper GI	35 (17%)	53 (20%)	22 (19%)	0.87	.78
Genitourinary	69 (34%)	88 (33%)	35 (31%)	0.89	.65
Head and neck	43 (21%)	61 (23%)	27 (25%)	0.98	.72
Thoracic	8 (4%)	7 (2%)	5 (4%)	1.45	.45
Others	8 (4%)	8 (3%)	1 (1%)	0.93	.47

The table lists demographic details, past medical history including cardiovascular (CVA and TIA) and respiratory conditions (COPD), outcomes including mortality rates and readmission rates, and medications used. Data are presented as the number and percentage of patients positive (Y) or negative (N) to each parameter. χ² statistic and *P* value columns report results of the χ² tests assessing differences between groups.

Abbreviations: ACE indicates angiotensin-converting enzyme; CABG, coronary artery bypass grafting; COPD, chronic obstructive pulmonary disease; CVA, cardiovascular accident; GI, gastrointestinal; MI, myocardial infarction; POMS, Postoperative Morbidity Score; TIA, transient ischemic attack.

Operation types were colorectal (20%), upper gastrointestinal (18%), genitourinary (33%), head and neck (23%), thoracic (4%), and other (2%). Patients were observed postoperatively in the postoperative care unit/HDU (POCU; 64%), ward (29%), and ICU (7%). Median numbers of POMS-positive domains on postoperative days 3, 5, and 7 were 3, 2, and 2, respectively.

### Population Demographics

Tables 1 and 2 detail the demographic and medical histories of the study cohort. Significant variations in age and BMI were observed between the POMS 0 to 1 group and the POMS 2 and POMS 3 to 6 groups. No significant difference was noted between POMS 2 and POMS 3 to 6. Laboratory and blood pressure values were consistent across all groups. The POMS 2 and POMS 3 to 6 groups to the POMS 0 to 1 group, but had a greater prevalence of previous myocardial infarction, diabetes, and arthritis, and were more frequently prescribed statins and beta blockers. Both POMS 2 and 3 to 6 groups had higher ASA scores (*P* < .001) and lower DASI scores (*P* < .001).

### Mortality and Readmission

Thirty-day mortality increased in line with the POMS category: (POMS 0–1: 0.5%, POMS 2: 1.49% and POMS 3–6: 5.31%; *P* = .04). One-year mortality rates also correlated (POMS 0–1: 1.47%, POMS 2: 2.99%, POMS 3–6: 8.85%, *P* = .01). Length of stay (LOS, days) rose with increasing morbidity (POMS 0–1: 9 [7–15]; POMS 2: 11 [8–16]; POMS 3–6: 13 [9–20]; *P* < .001; Tables 1 and 2).

### Time Series CPET Analysis

Oxygen consumption (VO_2_/kg) data for each individual test were plotted as a time series for the 3 POMS groups (Supplemental Digital Content 2, Supplementary Figure 1, http://links.lww.com/AA/F257). There was a difference in oxygen consumption profiles during exercise between the lower postoperative morbidity survey group (POMS 0–1) and the other 2 increasing morbidity groups (POMS 2 and POMS 3–6), with the former demonstrating higher peak values, indicative of better cardiorespiratory performance. When comparing the POMS 2 and POMS 3 to 6 groups, a significant overlap was observed, making it challenging to differentiate between the 2 groups based on VO_2_/kg curves alone. Despite a general trend indicating that a lower VO_2_ peak was associated with a higher risk of complications, the considerable overlap seen between the POMS 2 and 3 to 6 groups suggests that oxygen consumption values have limited utility in separating the prediction of moderate versus severe complications (Table 1).

### CPET Analysis

All CPET analyses were conducted using a standardized protocol involving an average ramp of 10 W/min, with no discernible differences in protocol adherence across groups.

CPET metrics are reported in Table 1. Differences between postoperative morbidity groups are described. Resting oxygen pulse and oxygen consumption metrics were different across POMS 0 to 1 vs POMS 2 and POMS 3 to 6 (both *P* < .05 vs POMS 0–1). No significant differences were observed between POMS 2 and POMS 3 to 6 groups. RER and VE/VCO_2_ values also differed significantly between POMS 0 to 1 and POMS 2 groups (Table 1).

VO_2_ at the ventilatory AT consistently decreased from the POMS 0 to 1 group to POMS 2 to the POMS 3 to 6 (both *P* < .05 vs POMS 0 to 1). Conversely, VE/VCO_2_ values increased from POMS 0 to 1 to POMS 2 and POMS 3 to 6 (both *P* < .001 vs POMS 0–1). The POMS 2 and POMS 3 to 6 groups did not exhibit any significant difference.

At peak VO_2_, significant differences in most parameters (including HR, oxygen pulse, oxygen consumption, and VE/VCO_2_ (all *P* < .05), were noted between POMS 0 to 1 and POMS 2 and POMS 3 to 6. No differences were noted between POMS 2 and POMS 3 to 6 groups.

### Regression Analysis

Polynomial curves and regression analysis are shown in Figure 2, to analyze the relationship between exercise test (CPET) values (specifically VO_2_ and VE/VCO_2_) and CHOox and increasing postoperative morbidity. A moderate association was noted between decreasing peak oxygen consumption (VO_2_) and increased postoperative morbidity, (*P* < .05, coefficient of determination *R*^2^ = 0.42). A plateau was seen in VO_2_ peak values at or above a POMS category of 2. This plateau implies that beyond a POMS category of 2, a saturation point is reached in the relationship between peak VO_2_ and increasing morbidity. Conversely, the association between VE/VCO_2_ values and cumulative POMS scores was poor (*P* < .05, *R*^2^ = 0.13). Ultimately, a moderate association was observed between decreasing peak CHOox and cumulative postoperative morbidity (POMS) (*P* < .05, *R*² = 0.63). Considerable overlap was noted between all POMS classes, indicating substantial variability in CPET values within each POMS group.

### Metabolic Flexibility

Table 3 and Figure 2 describe metabolic responses (substrate utilization) during exercise across the different POMS groups. Substrate utilization was assessed through measurement of the area under the curve (AUC) during a time-normalized exercise test to enable assessment of the relative contribution of fat and CHOox to the overall test performance.

**Table 3.** CHOox and FATox at Various Stages Including Maximum, AT, and Recovery Phases, and Relationship to the Cumulative POMS Categories: 0 to 1, 2, and 3 to 6

	POMS 0–1 median (IQR), n = 204	POMS 2 median (IQR), n = 268	POMS 3–6 median (IQR), n = 113	ANOVA *P* value	POMS 0–1 vs POMS 2	POMS 0–1 vs POMS 3–6	POMS 2 vs POMS 3–6
Fuel oxidation (g/min)							
FATox AUC	826 (578–1147)	658 (448–922)	608 (414–845)	<.001	++	++	--
FATox peak	0.22 (0.17–0.30)	0.19 (0.14–0.24)	0.16 (0.12–0.23)	<.001	++	++	--
FATox baseline	0.06 (0.03–0.08)	0.04 (0.02–0.06)	0.04 (0.02–0.06)	<.001	++	++	--
CHOox AUC	10,277 (7773–13,358)	8356 (6548–10377)	6696 (4739–9392)	<.001	++	++	++
CHOox peak	3.3 (2.4–4.3)	2.4 (1.9–3.0)	1.8 (1.2–26)	<.001	++	++	++
CHOox baseline	0.06 (0.02–0.12)	0.06 (0.02–0.13)	0.05 (0.02–0.11)	.655	--	--	--
CHOox recovery	0.92 (0.66–1.62)	0.88 (0.59–1.20)	0.70 (0.38–1.05)	<.001	++	++	--

Parameters analyzed are fuel oxidation (measured in g/min) for both CHOox and FATox at different stages including AUC, peak, baseline, and recovery. Values are presented as median and IQR for each POMS category. The last 4 columns provide the ANOVA *P* value and the results of pairwise comparisons between the POMS categories, indicating statistical significance (++) or not (--).

Abbreviations: ANOVA, analysis of variance; AT, aerobic threshold; AUC, area under the curve; CHOox, carbohydrate oxidation; FATox, fatty acid oxidation; IQR, interquartile range; POMS, Postoperative Morbidity Score.

The lowest postoperative morbidity group (POMS 0–1) exhibited significant increases in overall fat oxidation when compared with higher postoperative morbidity (POMS 2 and POMS 3–6; both *P* < .001 vs POMS 0–1). POMS 0 to 1 also demonstrated a higher absolute peak FATox, compared to the other 2 groups (*P* < .001 vs POMS 0–1).

Significant decreases were reflected in the median CHOox AUC values. These were highest in the POMS 0 to 1 group followed by POMS 2 and POMS 3 to 6, with differences being statistically significant (*P* < .001), including between POMS 2 and POMS 3 to 6 groups.

Regarding CHOx at peak exercise (CPET peak), there was a noticeable decrease in oxidation rates across the POMS groups from lowest morbidity to highest. The highest oxidation rate was observed in the POMS 0 to 1 group compared to the POMS 2 group, and this further decreased in the POMS 3 to 6 group (all *P* < .001). CHOox in the recovery phase after exercise also showed a progressive fall from the POMS 0 to 1 group (all *P* < .001).

Figure 2 illustrates differences in metabolic crossover points between the POMS groups. There was a clear crossover point in fuel selection in the POMS 0 to 1 group where predominant fat oxidation switches to predominant CHOox as the intensity of the exercise increases. Progression from POMS 1 to POMS 2 and then POMS 3 to 6 illustrates the loss of this crossover point.

### Confounder Analysis

Confounder analysis (Supplemental Digital Content 1, Supplementary Table 1, http://links.lww.com/AA/F256) revealed that CHOox (AUC peak) demonstrated the strongest association with outcomes (β = −0.106, *P* = .0005). BMI and sex showed statistically significant but modest associations (*P* = .045 and *P* = .049, respectively). Several clinical variables approached statistical significance, including previous myocardial infarction (β = 0.213, *P* = .054), β-blocker use (β = 0.287, *P* = .061), and ASA grade (β = 0.234, *P* = .068). Operation severity demonstrated a notable effect (β = 0.224, *P* = .071), while current smoking showed a negative association approaching significance (β = −0.230, *P* = .074). Other cardiovascular risk factors, medications, and comorbidities showed no significant associations (all *P* > .10).

## DISCUSSION

We observed distinct differences in metabolic responses between patients with varying degrees of postoperative morbidity. Patients with lower postoperative morbidity demonstrated greater flexibility in substrate selection, with higher rates of fat oxidation during the early stages of exercise. In contrast, patients with higher morbidity exhibited reduced fat oxidation and increased reliance on carbohydrates for energy production. At peak exercise, patients with lower postoperative morbidity were able to oxidize significantly more carbohydrates than those who went on to develop morbidity, suggesting greater metabolic flexibility.

As described in other studies,^10,27–30^ traditionally reported CPET metrics such as peak oxygen consumption and AT^10,25–28^ could also distinguish between patients who did or did not develop postoperative morbidity. However, these metrics were unable to identify between those patients who developed morbidity across a few or multiple POMS domains on Postoperative day 3. Given that categorization into low, moderate, and high POMS-defined morbidity is also associated with differences in 1-year mortality,^31^ the ability to discriminate between groups is of potential importance. Furthermore, given that metabolic inflexibility is a potentially modifiable risk factor, early identification could allow for interventions such as dietary modification or exercise training to modify perioperative risk.

Patients in the POMS 2 and 3 to 6 groups had body composition, and medical and drug histories consistent with an increased risk of metabolic syndrome, as well as a greater prevalence of diabetes. These morbidities alone increase the likelihood of metabolic inflexibility during aerobic exercise. Lower CRF is common in patients with type 2 diabetes, metabolic syndrome, and obesity.^32,33^ Middle-aged and older overweight obese patients often demonstrate impaired insulin sensitivity and lower muscle glycogen content. This affects the ability to increase fat oxidation during submaximal exercise and to switch from fat to CHOox when moving from rest to exercise of increasing intensity.^34^ This inability to effectively regulate fat and CHOox during aerobic exercise could limit the ability to supply sufficient energy at higher levels of aerobic exercise. These findings are congruent with the observation that the maximal fat oxidation of normal-weight subjects is significantly greater than that of overweight and obese subjects per unit of metabolically active tissues, independent of age, sex, or CRF.

In the perioperative period, multiple factors influence both energy supply and metabolic demand. Tissue trauma secondary to the surgical insult drives increased metabolic demand. Fasting and delayed postoperative feeding will deplete glycogen reserves while insulin resistance secondary to the inflammatory response will further reduce metabolic flexibility and the ability to access energy stores, both in terms of glycogen and lipid. The increased metabolic demand of the early postoperative period may reflect that of submaximal exercise with established metabolic inflexibility resulting in reduced physiological resilience to surgical trauma.^13,35^ Both the moderate and severe morbidity groups in this study reported median average BMI’s that fell within the “overweight” category. The POM 0 to 1 group median also closely bordered on overweight, with the IQR incorporating many of the overweight categories. While BMI serves as a quick indicator of patient risk, the current literature highlights its limitations as a sole factor in assessing risk, as it fails to provide insights into body composition (eg, visceral adiposity or intramuscular adipose tissue), which are key to understanding metabolic flexibility.^36,37^ A potential way to address this limitation could be the use of new indices, such as the Body Roundness Index, which are showing promising results and may be recommended for future clinical practice.^38,39^

This study is subject to limitations. The retrospective observational design used data from a prospectively collected institutional database. This approach allowed the inclusion of a large cohort of patients but is susceptible to biases and unmeasured confounders that may have influenced the results. The incorporation of unmeasured confounders can result in model misspecification. Future work should seek to externally validate the findings of this study and incorporate additional parameters to refine the relative contributions of metabolic features derived from CPET to other parameters that might be predictive of the development of morbidity. The data are derived from a single institution, potentially limiting generalizability to other health care settings. The metabolic flexibility data does however reflect those reported in other nonsurgical studies, and the mechanism has biological plausibility. Patients included in this study may be subject to selection bias since patients who undergo perioperative exercise testing may differ systematically from those who do not, again potentially affecting the external validity of the findings. This is mitigated somewhat by CPET referral being routinely protocolized in most patients undergoing complex major surgery in our institutional database, reducing selection bias. The use of CPET to assess substrate utilization is subject to some potential inaccuracies. Coefficients of variation for measurement of crossover point and maximum lipid oxidation have been reported ranging from 7% to 21% and 7% to 12%, respectively. The most comprehensive study assessing this outcome reported high relative reliability for the crossover plot (6% and 7%, respectively), although this study was performed in a more homogenous and fitter population than ours.^40–43^ This study assessed POMS-defined morbidity as the primary outcome measure, as well as mortality and length of hospital stay. While these outcomes are clinically relevant, they represent only a proportion of postoperative outcomes. POMS classified by the number of positive domains in this study was associated with increased mortality and hospital length of stay but is limited by the nature of POMS as a nonweighted binary outcome measure. Despite efforts to control confounders through statistical analysis, confounding variables may play a role. Socioeconomic status, nutritional status, and intraoperative management practices could potentially influence both metabolic flexibility and perioperative outcome.

Metabolic flexibility is a complex physiological phenomenon influenced by multiple factors. While an accepted method of describing substrate selection during exercise, metabolic flexibility derived from maximal exercise testing itself can be open to interpretation and may not directly reflect actual substrate utilization.^14^ Anaerobic versus aerobic glycolysis before and after the ventilatory AT, as well as acid buffering via the Henderson-Hasselbalch equation, will affect the derivation of carbohydrate metabolism beyond the AT. Despite this, the relationships described in this study reflect those described in other contexts in healthy individuals, patients with a variety of medical conditions, and those with known metabolic syndrome.

Beyond metabolic syndrome and diabetes, metabolic inflexibility has mechanistic relevance to both exercise performance and outcomes in both aging and sarcopenia. Patients in the POMS groups defined in this study were of increasing age with increasing total positive POMS domains on day 3 after surgery. At first glance, this alone might explain the differences in outcomes reported across groups. However, while likely that the differences in metabolic flexibility seen with increasing morbidity may partly be associated with age, the mechanisms through which metabolic inflexibility influences outcome are likely to be of more relevance than age by itself as an exposure. Age-related progressive loss of skeletal muscle mass and strength (sarcopenia) are associated with adverse postoperative outcomes,^44^ including an increased risk of complications and mortality up to 5 years. An elevated BMI (noted in many patients included in this study) does not preclude sarcopenia which may be present in up to 50% of obese patients.^45,46^ Compared with nonsarcopenic patients, sarcopenic patients have a reduced ability to utilize fat for fuel during steady-state aerobic exercise, and a decreased ability to use carbohydrate during anaerobic exercise.^47^

These findings suggest that metabolic inflexibility may contribute to the development of postoperative complications by impairing the body’s ability to adapt to increased metabolic demands. Moreover, they highlight the potential utility of CPET as a tool for assessing metabolic flexibility and identifying patients at higher risk of postoperative morbidity. By targeting metabolic inflexibility through interventions such as prehabilitation, it may be possible to improve perioperative outcomes and enhance patient recovery.

Future work should aim to prospectively confirm the relationship between metabolic flexibility, exercise capacity, and perioperative outcomes. It should also explore underlying mechanisms linking metabolic flexibility to perioperative outcomes using experimental models and translational research approaches. These will include pathways involved in metabolic regulation, immunometabolism and inflammation, oxidative stress, and tissue repair to identify potential therapeutic targets for intervention. Patient-centered outcomes research should evaluate the impact of metabolic inflexibility as an exposure on quality of life and recovery after surgery, as well as its impact on longer-term survival. Future studies should incorporate patient-reported outcomes and qualitative assessments.

## DISCLOSURES

**Conflicts of Interest:** None. **Funding:** None. **This manuscript was handled by:** Tong J. Gan, MD.

## Supplementary Material
