# Supplementary material for: Metabolic Flexibility as a Candidate Mechanism for the Development of Postoperative Morbidity
Source: Anesth Analg. 2025 Apr 2;141(5):1078–88. doi: 10.1213/ANE.0000000000007494 (PMC12509443; doi:10.1213/ANE.0000000000007494)
Supplement: Supplementary file 1 [file ane-141-1078-s001.docx]

**Supplementary Table 1: Regression Analysis of Confounders Impacting Patient Outcomes**

This table presents the results of a regression analysis evaluating the relationship between demographic, clinical, and laboratory test confounders and patient outcomes. All variables were treated as linear in the confounder analysis. The coefficients (Coef.) indicate the direction and magnitude of the association, while Confidence Intervals (CI) reflect the variability of the estimates. The p-values assess the statistical significance of each confounder, with p-values less than 0.05 denoting significant associations. Key variables include carbohydrate oxidation (CHOox AUC Peak), Body Mass Index (BMI), sex, previous myocardial infarction (MI), beta blocker usage, American Society of Anesthesiologists (ASA) scores, operation severity, and various laboratory test results (hemoglobin [Hb], sodium [Na], potassium [K]). Comorbidities such as smoking, asthma, diabetes, chronic obstructive pulmonary disease (COPD), and others are also analyzed to understand their influence on patient outcomes. Significant results are highlighted for clinical relevance, particularly emphasizing the impact of carbohydrate oxidation on patient health outcomes.

| **Column1** | **Coef.** | **CI lower** | **CI upper** | **p-value** |
| --- | --- | --- | --- | --- |
|  |  |  |  |  |
| CHOox AUC Peak | -0.106 | -0.164 | -0.047 | 0.0005 |
| BMI | 0.035 | 0.023 | 0.046 | 0.045 |
| Sex | 0.158 | 0.001 | 0.314 | 0.049 |
| Previous MI | 0.213 | 0.028 | 0.397 | 0.054 |
| Beta blocker | 0.287 | 0.043 | 0.530 | 0.061 |
| ASA | 0.234 | 0.128 | 0.339 | 0.068 |
| Operation severity | 0.224 | 0.092 | 0.355 | 0.071 |
| Smoking | -0.230 | -0.482 | 0.022 | 0.074 |
| Asthma | 0.254 | 0.016 | 0.491 | 0.087 |
| Duke Activity Status Index | -0.004 | -0.007 | 0.001 | 0.089 |
| White blood cell count | 0.019 | -0.004 | 0.042 | 0.119 |
| Statins | 0.125 | -0.043 | 0.293 | 0.147 |
| Na | -0.015 | -0.038 | 0.008 | 0.201 |
| Coronary stent | -0.198 | -0.513 | 0.117 | 0.219 |
| K | -0.097 | -0.251 | 0.057 | 0.221 |
| Const | 1.912 | -1.555 | 5.379 | 0.280 |
| Nitrates | -0.213 | -0.608 | 0.182 | 0.292 |
| Diabetes | 0.096 | -0.094 | 0.286 | 0.322 |
| Arthritis | -0.076 | -0.242 | 0.090 | 0.375 |
| Urea | 0.007 | -0.008 | 0.022 | 0.375 |
| COPD | 0.095 | -0.173 | 0.363 | 0.485 |
| Creatinine | 0.001 | -0.001 | 0.002 | 0.494 |
| Age | 0.001 | -0.004 | 0.006 | 0.624 |
| Cardiac failure | 0.059 | -0.191 | 0.309 | 0.645 |
| Angina | 0.081 | -0.299 | 0.461 | 0.675 |
| Pulmonary embolism | -0.091 | -0.530 | 0.348 | 0.683 |
| ACE inhibitor | -0.032 | -0.228 | 0.163 | 0.749 |
| Peripheral vascular disease | 0.069 | -0.389 | 0.527 | 0.767 |
| Cerebrovascular Accident or Transient Ischemic Attack | -0.050 | -0.383 | 0.283 | 0.768 |
| Pulmonary fibrosis | 0.089 | -0.626 | 0.804 | 0.806 |
| Hemoglobin | 0.000 | -0.003 | 0.003 | 0.876 |
| CABG | -0.013 | -0.434 | 0.408 | 0.951 |
| Hypertension | -0.004 | -0.174 | 0.166 | 0.965 |
