# Supplementary material for: Metabolic Flexibility as a Candidate Mechanism for the Development of Postoperative Morbidity
Source: Anesth Analg. 2025 Apr 2;141(5):1078–88. doi: 10.1213/ANE.0000000000007494 (PMC12509443; doi:10.1213/ANE.0000000000007494)
Supplement: Supplementary file 2 [file ane-141-1078-s002.pptx]

## Slide 1
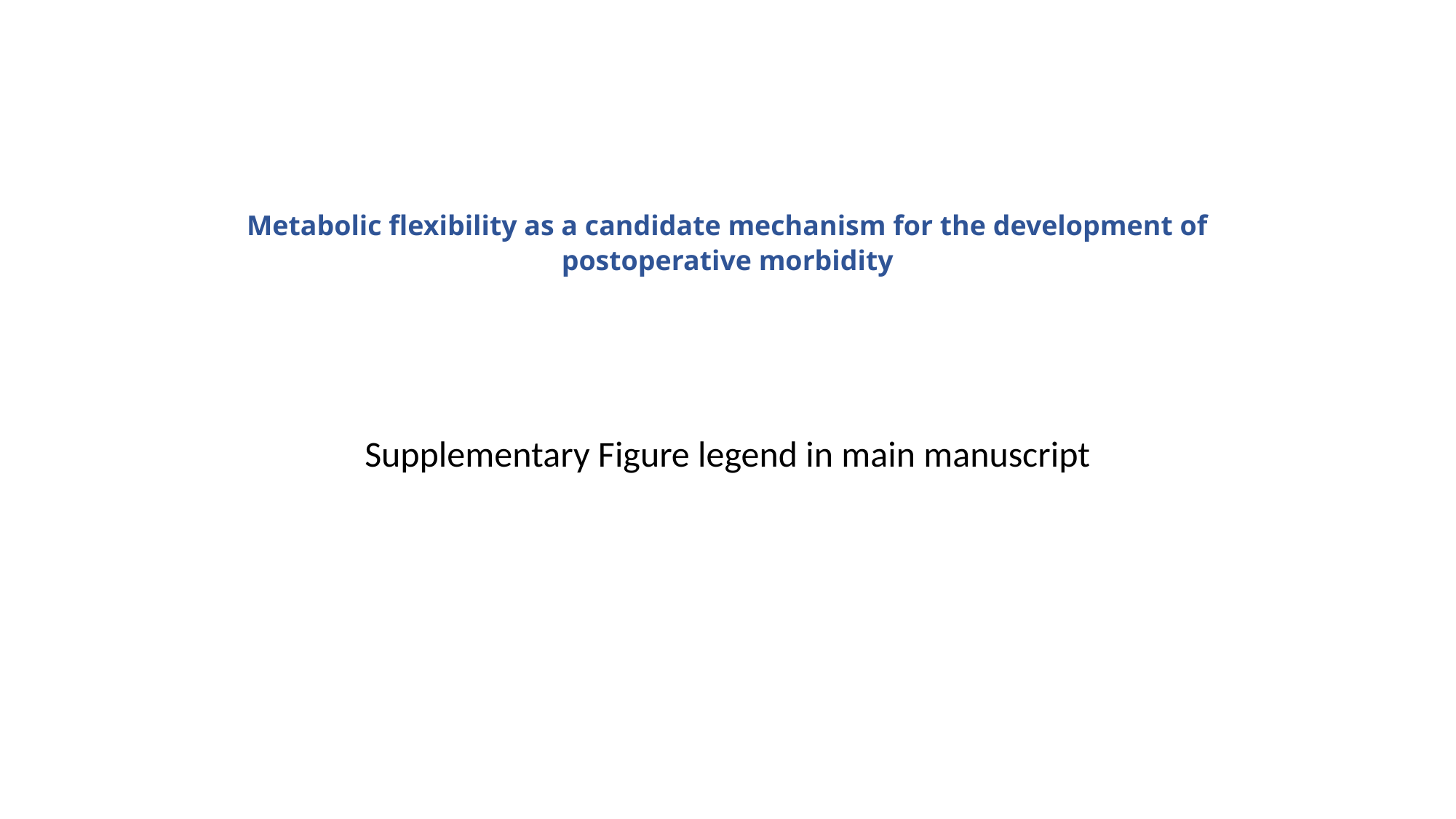

# Metabolic flexibility as a candidate mechanism for the development of postoperative morbidity
Supplementary Figure legend in main manuscript

## Slide 2
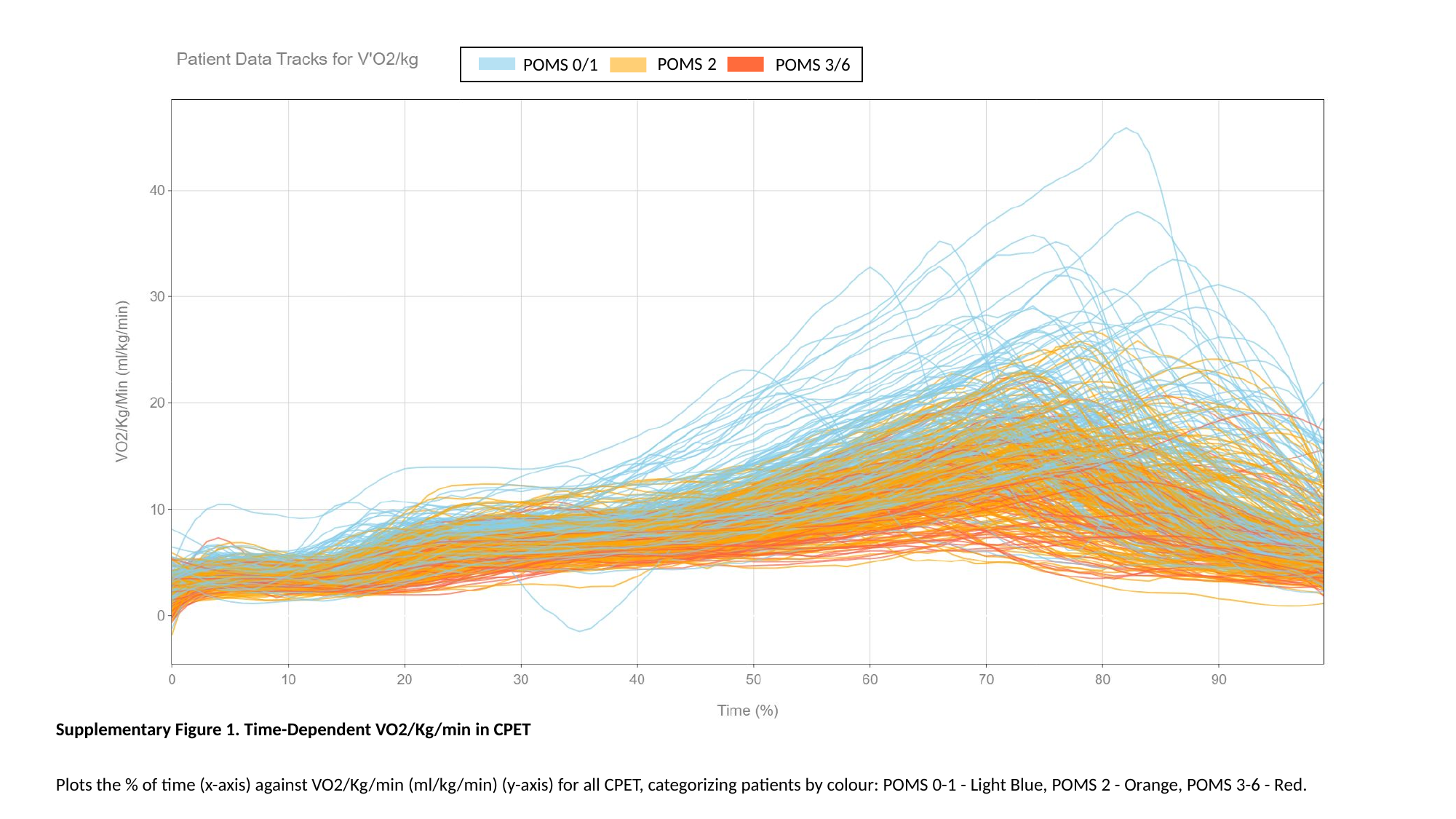

POMS 2
POMS 3/6
POMS 0/1
Supplementary Figure 1. Time-Dependent VO2/Kg/min in CPET
Plots the % of time (x-axis) against VO2/Kg/min (ml/kg/min) (y-axis) for all CPET, categorizing patients by colour: POMS 0-1 - Light Blue, POMS 2 - Orange, POMS 3-6 - Red.
